# A Case Study on Reinforcing Asphalt Pavement Using Sensing Geogrid Based on Fiber Bragg Grating

**DOI:** 10.3390/ma19132749

**Published:** 2026-06-27

**Authors:** Jian Liu, Yanlei Bi, Qiaoyi Li, Guangqing Yang, Peng Xu

**Affiliations:** 1 Cangzhou Qugang Expressway Construction Co., Ltd., Cangzhou 061000, China; 2 School of Traffic and Transportation, Shijiazhuang Tiedao University, Shijiazhuang 050043, China; 3 School of Urban Geology and Engineering, Hebei GEO University, Shijiazhuang 050030, China; 4 Key Laboratory of Roads and Railway Engineering Safety Control of Ministry of Education, Shijiazhuang Tiedao University, Shijiazhuang 050043, China; 5 Hebei Engineering Research Center on Application of Geosynthetics, Shijiazhuang Tiedao University, Shijiazhuang 050043, China; 6 School of Civil Engineering, Shijiazhuang Tiedao University, Shijiazhuang 050043, China; 7 State Key Laboratory of Mechanical Behavior and System Safety of Traffic Engineering Structures, Shijiazhuang Tiedao University, Shijiazhuang 050043, China

**Keywords:** asphalt pavement, reflective cracking, fiber Bragg grating, sensing geogrid

## Abstract

When traditional geogrids are used to mitigate reflective cracks in asphalt pavement, it is difficult to monitor the internal state of the pavement and the strain of the geogrid in real time. This study proposes a sensing geogrid based on warp-knitting technology, where fiber Bragg grating (FBG) sensors are embedded into the geogrid through the weaving process, enabling it to possess both reinforcement and strain-sensing functions. The sensing geogrid was calibrated through laboratory tensile tests, and field monitoring was conducted to obtain optical signal variation data at various stages during asphalt pavement paving, as well as the deformation of the geogrid at different measurement points in each stage. The results indicate that the weaving process did not damage the FBG sensors, and the sensing geogrid exhibited good optical signal performance and normal signal acquisition during the production and transportation stages. The strain of the FBG sensors and the geogrid showed a linear correlation, with a correlation coefficient of 845 με/nm, demonstrating good deformation compatibility between them. Field monitoring confirmed that the sensing geogrid has good construction adaptability and can perceive fluctuations in optical signals and deformation of the geogrid during the construction process. Specifically, significant deformation of the geogrid occurred during the construction of the asphalt-treated base (ATB-25) and bottom layers, accompanied by substantial fluctuations in optical signals due to construction machinery. In contrast, signal fluctuations were smaller during the construction of the middle and surface layers, with the influence depth of construction machinery being approximately 22 cm. Compared to ordinary road sections, the deflection basin curve of the reinforced section was gentler, and the maximum deflection was reduced by approximately 41%. This study confirms the feasibility of the sensing geogrid and provides a valuable reference for its application in road engineering.

## 1. Introduction

Semi-rigid base asphalt pavements are prone to various types of distress due to factors such as temperature, traffic loads, and water, which compromise their structural integrity and reduce their service performance and lifespan [1,2,3]. Furthermore, as semi-rigid base materials are sensitive to changes in temperature and humidity, they are susceptible to shrinkage cracking under low-temperature conditions. These cracks propagate upward into the surface layer, forming reflective cracks that severely shorten the pavement’s service life [4,5,6]. To enhance the durability of semi-rigid base asphalt pavements, researchers have employed geosynthetic materials (geotextiles, geogrids, etc.) to reinforce them, aiming to improve the structural performance and extend the service life of the pavement [7,8,9].

Kumar et al. [10,11,12] conducted tensile strength tests, repeated load tests, and asphalt beam fatigue tests to investigate the improvement effects of different types of materials on the performance of asphalt overlay structures and to reveal the underlying mechanisms. All geosynthetic materials significantly enhanced the crack resistance, deformation resistance, and fatigue resistance of the asphalt overlay, with geogrids generally providing better reinforcement effects than geotextiles. Khodaii et al. [13] studied the influence of geogrid position on delaying reflective cracking in asphalt overlays through laboratory fracture tests. Their research showed that placing the geogrid at one-third of the overlay thickness from the bottom yielded the optimal effect. Nejad et al. [14,15] investigated the influence of various factors on reflective cracking in asphalt overlays using response surface methodology and cyclic load tests. The results indicated that the fatigue life of asphalt overlays is significantly affected by temperature, and geosynthetic materials with higher moduli perform better in delaying reflective cracking, with geogrids demonstrating a noticeably superior reinforcement effect compared to geotextiles. Ling et al. [16] proposed a new method for evaluating reflective cracking in pavements and verified its feasibility through laboratory tests. Khan et al. [17] evaluated the strain response of asphalt overlays reinforced with geosynthetic materials by combining laboratory tests with full-scale tests. The results demonstrated that asphalt overlays reinforced with geosynthetics exhibit superior crack resistance and can withstand greater tensile strain. Zarei et al. [18] explored the relationship between the performance of geogrid-reinforced asphalt overlays and geogrid modulus through finite element simulations. The results showed that the performance of asphalt overlays is closely related to the geogrid modulus, with high-modulus geogrids inducing more significant mechanical responses in the pavement.

The aforementioned studies indicate that geogrids can effectively enhance pavement performance and delay crack initiation in asphalt pavements. However, in practical engineering, after a geogrid is installed in an asphalt pavement, the true mechanical response of its internal structure is difficult to monitor in real time. In recent years, fiber optic sensors have been introduced into the field of structural health monitoring for civil engineering due to their excellent properties [19,20,21]. Therefore, researchers have attempted to combine fiber optic sensors with geogrids. By perceiving the deformation and stress response of the geogrid itself in real time, they aim to thereby indirectly reflect changes in the mechanical state inside the measured matrix [22,23,24].

Zhong et al. [25] developed a sensing geogrid integrating reinforcement and real-time strain monitoring functions by combining fiber Bragg grating (FBG) sensing technology with three-dimensional (3D) printing. They established a theoretical strain transfer model considering the interaction between the optical fiber, adhesive layer, and geogrid and conducted tensile tests to verify the model’s reliability. Wang et al. [26] addressed the issue of cumulative error in displacement reconstruction for sensing geogrids integrated with FBG sensors by proposing a segmented displacement reconstruction method to improve measurement accuracy. Simulation and experimental verification showed that this method can significantly reduce displacement reconstruction errors and enhance the sensing accuracy of the sensing geogrid. Hong et al. [22] used epoxy resin to bond FBG sensors to a geogrid and revealed the strain transfer mechanism between them through tensile tests. The results indicated that the bonding width and length are the dominant factors affecting the average strain transfer coefficient. Yu et al. [27] embedded fiber optic sensors beneath a geogrid and conducted field monitoring on a highway test section. Based on one year of temperature and strain tracking, a quantitative regression relationship was established between base layer temperature, strain, and atmospheric temperature. Imjai et al. [28] placed fiber optic sensors above geosynthetics (geotextile and geogrid) and performed full-scale tests to monitor the stress and strain in the geosynthetics and asphalt pavement structure.

The above research indicates that the integration of fiber optic sensors with geogrids primarily relies on manual adhesive bonding. Due to the uneven adhesive application inherent in manual operation, a shear lag effect is prone to occur at the bonding interface between the sensor and the geogrid. Furthermore, existing studies have largely focused on laboratory tests and have not addressed the adaptability issues between fiber optic sensors and geogrids in actual construction processes. To overcome these limitations, this study proposes a sensing geogrid based on a warp-knitting process, where FBG sensors are embedded into the geogrid during weaving, endowing it with both reinforcement and strain-sensing capabilities. The sensing performance and construction adaptability of the sensing geogrid were validated through laboratory tests and on-site monitoring during the construction of the Qugang Expressway in Hebei Province. Installed at the interface between the asphalt-treated base (ATB-25) and the cement-stabilized macadam (CSM) base, the sensing geogrid successfully enabled dynamic response monitoring during construction compaction. Its reinforcement effectiveness was further verified through on-site falling weight deflectometer (FWD) tests.

## 2. Materials and Methods

### 2.1. Materials

**Figure 1:**
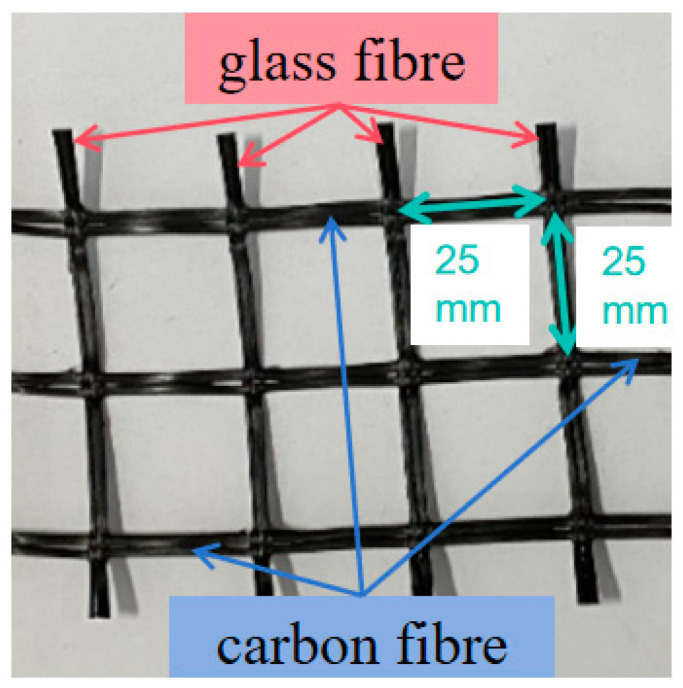
Composite geogrid woven from glass fiber/carbon fiber.

**Figure 2:**
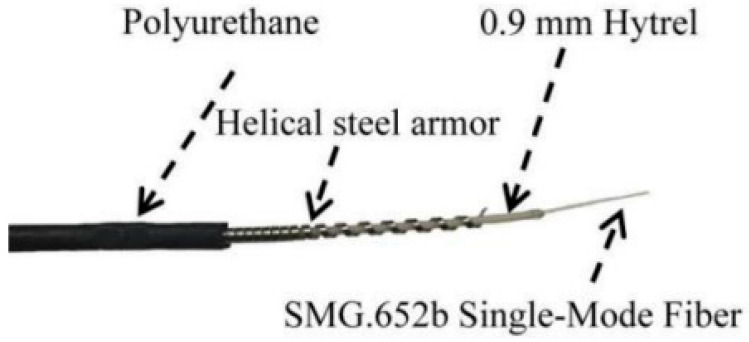
Armored Bragg fiber optic sensor [29].

The geogrid used in this study is a composite geogrid woven from glass fiber/carbon fiber (Figure 1). Its transverse ribs consist of 12K carbon fiber, and the longitudinal ribs are made of 1100 tex glass fiber. The physical and mechanical properties of the composite geogrid are presented in Table 1. The selected optical fiber sensor is an armored FBG sensor (Figure 2) produced by Nanjing Nanzhi Advanced Optoelectronic Integration Technology Research Institute Co., Ltd. (Nanjing, China). The sensor cable has a diameter of 2.5 mm, a wavelength range of 1528 nm to 1568 nm, a total length of 80 m, and a sensing point spacing of 1 m.

### 2.2. Fabrication of the Sensing Geogrid

**Figure 3:**
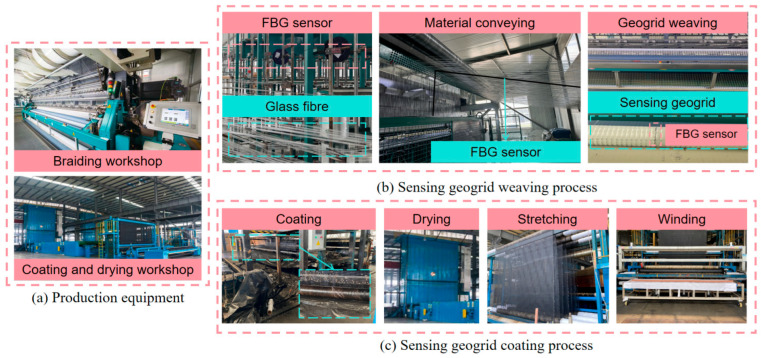
Production process of the sensing geogrid [29].

**Figure 4:**
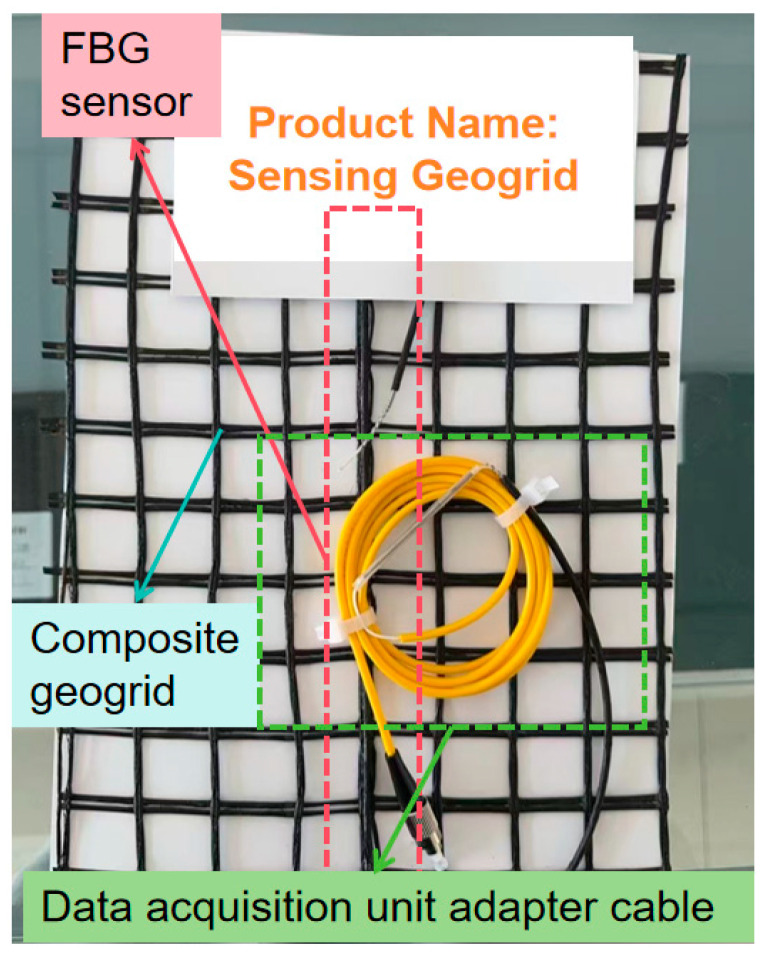
Finished sensor-equipped geogrid.

Before the optical cable is embedded into the sensing geogrid, the armored optical fiber sensors must be interrogated. During the manufacturing process, the armored optical fiber sensors are arranged along the longitudinal direction of the composite geogrid. One bundle of glass fibers in the knitting process is removed and replaced with the armored optical fiber sensor. The FBG sensor is then slowly introduced into the knitting system under mechanical traction. As the knitting process proceeds, the sensor is gradually embedded within the geogrid structure. The sensing geogrid is coated with SBS-modified asphalt. Subsequently, the coated sensing geogrid undergoes drying and curing treatment, and tension is applied in both the longitudinal and transverse directions to uniformly tighten the fiber bundles and improve the structural integrity. Finally, the finished product is rolled and packaged. The manufacturing process of the sensor-equipped geogrid is shown in Figure 3, and the finished sensor-equipped geogrid is shown in Figure 4.

### 2.3. Sensing Principle of the Sensing Geogrid

When the sensing geogrid is subjected to external loads, local strain is generated within its structure. This strain is captured in real-time by the embedded fiber optic sensor and converted into a corresponding shift in wavelength. By monitoring the wavelength shift of the sensor, the deformation location and strain distribution of the sensing geogrid can be back-calculated, thereby indirectly reflecting the mechanical response inside the asphalt mixture. Specifically, the wavelength shift Δλ of the FBG sensor and the axial strain ε of the optical fiber satisfy the following linear relationship, as shown in Equation (1):

(1) $${∆\lambda }_{BS}=\lambda_{B}\left(1-\rho_{\alpha }\right)∆\varepsilon =K_{\varepsilon }\lambda_{B}∆\varepsilon$$

where *Δλ_BS_* is the wavelength shift, *λ_B_* is the initial central wavelength, *Δε* is the axial strain, and *ρ_α_* is the photoelastic coefficient.

### 2.4. Performance Testing of the Sensing Geogrid

To evaluate the impact of the production process on the performance of the fiber optic sensors, a demodulation test was conducted on the sensing performance of the sensing geogrid using an optical fiber demodulator after its production was completed, and it had been wound and packaged. By comparing the response characteristics of the fiber optic sensors before and after implantation, as well as analyzing the integrity of their operational performance in both the wound state and after being unrolled and laid on-site, the stability of the sensor performance throughout the entire process can be effectively ensured.

### 2.5. Calibration of the Sensing Geogrid

To establish the strain–sensing response relationship of the sensing geogrid, this study calibrated the fiber optic sensor through a tensile test of the sensing geogrid. The sensing geogrid sample was installed on a GT1001-type electronic tensile testing machine (Tianjin Meitesi Testing Machine Factory, Tianjin, China), and the optical fiber demodulator was connected to the embedded fiber optic sensor to collect the sensor’s wavelength data in real time. By comparatively analyzing the strain curve of the geogrid obtained from the tensile test and the wavelength change curve output by the fiber optic sensor, a quantitative calibration relationship between the two was established. This provides a reliable sensing conversion basis for subsequent strain monitoring in practical engineering applications.

### 2.6. Installation of the Sensing Geogrid

**Figure 5:**
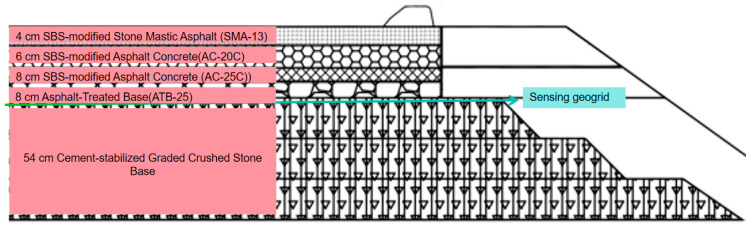
Pavement structure of the Qugang Expressway.

**Figure 6:**
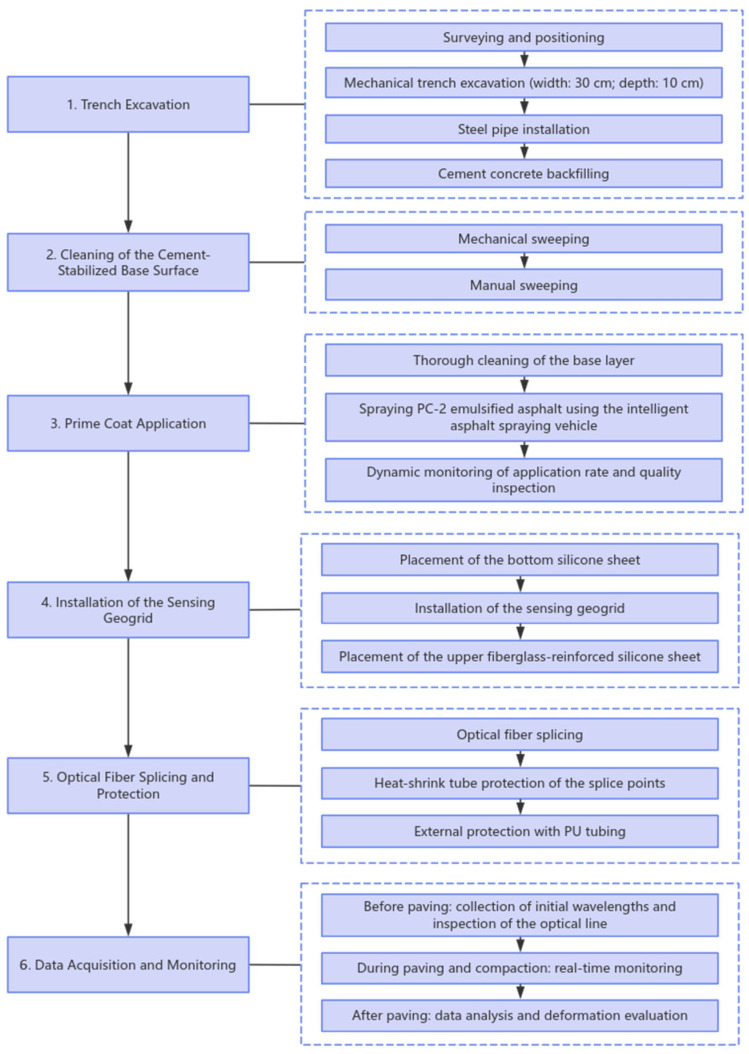
Installation process of the sensing geogrid.

**Figure 7:**
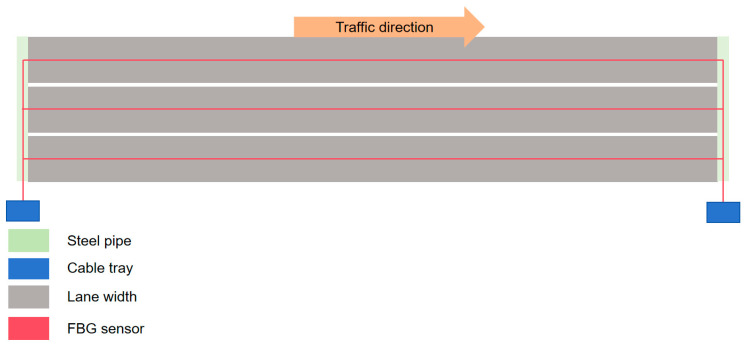
Sensor layout scheme.

The pavement structure of the Qugang Expressway is shown in Figure 5. The test section adopted a typical semi-rigid base asphalt pavement structure, consisting of a 4 cm SMA-13 upper surface layer, a 6 cm AC-20C intermediate surface layer, an 8 cm AC-25C lower surface layer, an 8 cm ATB-25 asphalt-treated base layer, and a 54 cm cement-stabilized crushed stone base. All asphalt layers were constructed using hot-mix asphalt mixtures. The sensing geogrid was installed between the ATB-25 layer and the cement-stabilized graded crushed stone base, with the sensors arranged along the traffic direction. To protect the optical fibers extending to the shoulder, trenches were excavated at the corresponding locations, and steel pipes were embedded and backfilled with cement concrete. Before installation, the surface of the cement-stabilized base was cleaned to ensure a level condition. Subsequently, a PC-2 emulsified asphalt prime coat was sprayed. Chip spreading was omitted in this section to avoid damage to the sensors. During installation, protective materials were placed in layers at the optical cable location, with a silicone sheet used as the bottom layer and a fiberglass-reinforced silicone sheet used as the upper layer. The joints were anchored using masonry nails. Finally, the initial wavelengths of the Fiber Bragg Grating (FBG) sensors were recorded before asphalt paving, and the connection status of the monitoring system was checked. The installation process of the sensing geogrid is shown in Figure 6, and the sensor layout scheme is shown in Figure 7.

Prior to asphalt pavement paving, the demodulator was connected to the sensing geogrid to record the optical signal changes at each monitoring point during construction. Compaction was carried out in three stages, totaling 5 to 6 passes. The first pass (initial compaction) utilized a double-drum vibratory roller (operating weight: 13 t, vibratory force: 126/84 kN). The second pass (intermediate compaction) employed a tire roller (operating weight: 30.3 t, ground contact pressure: 545 kPa). The third pass (final compaction) was again performed with the double-drum vibratory roller. By monitoring the wavelength changes at each monitoring point, the deformation of the geogrid at different locations was determined. Data were collected synchronously using this method during the paving of the different pavement structural layers.

### 2.7. FWD Test

**Figure 8:**
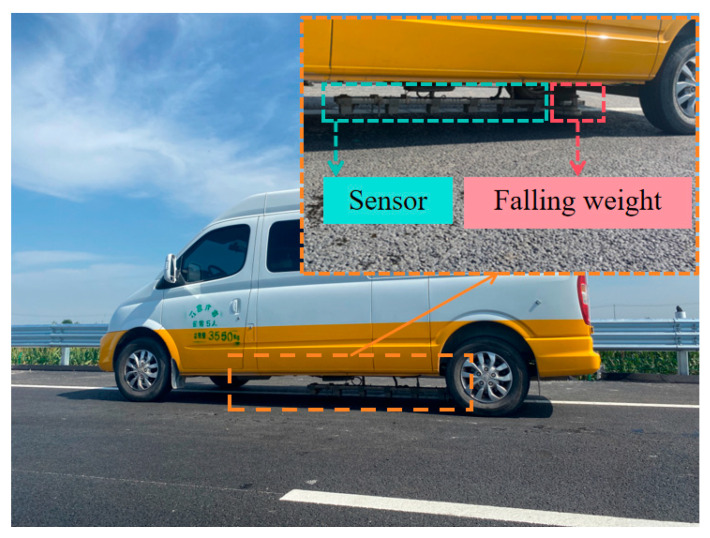
Pavement deflection basin test.

**Figure 9:**
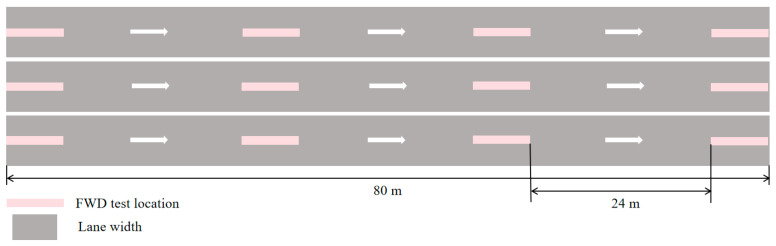
FWD test location.

To evaluate the treatment effectiveness of the sensing geogrid on reflective cracking in asphalt pavement, this study conducted FWD tests (Figure 8) after the paving of the Qugang Expressway was completed. The test compared the traffic lanes of a section reinforced with a sensing geogrid and a control section. Each section was tested 12 times, and the locations of the FWD tests are shown in Figure 9. To ensure the comparability of the test results, the control section and the sensing geogrid section were constructed with the same pavement structure, material composition, and construction procedures. The only difference between the two sections was the presence or absence of the sensing geogrid. An FWD was used to apply a dynamic load, while multi-point deflection data were collected simultaneously to generate the deflection basin. By comparing and analyzing differences in pavement surface deflection, the reinforcement effect of the geogrid on the pavement was evaluated, providing field-based mechanical evidence for long-term performance assessment.

## 3. Results

### 3.1. Performance Testing of the Sensing Geogrid

**Figure 10:**
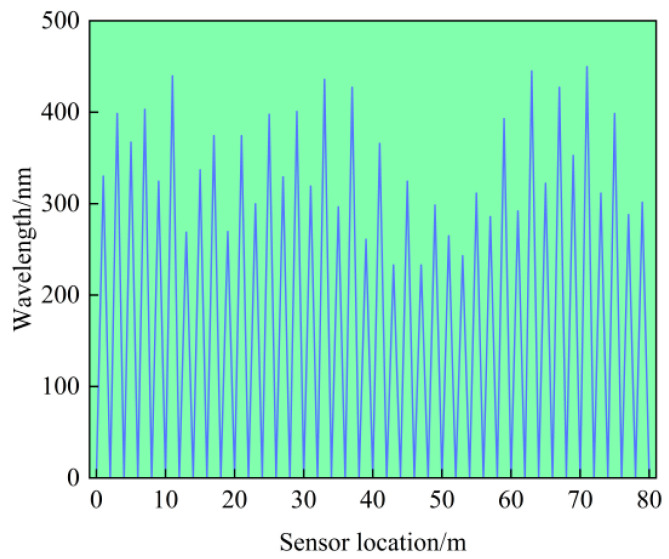
Optical signal distribution along the 80 m long FBG sensor before implantation.

**Figure 11:**
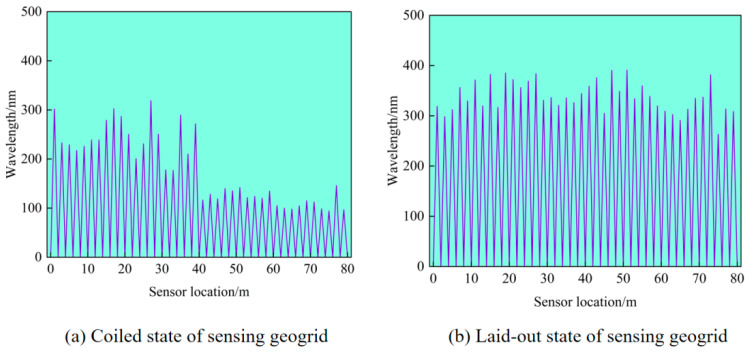
Optical signal distribution along the 80 m long FBG sensor under different states of the sensing geogrid.

To evaluate the performance stability of the fiber optic sensors throughout the entire process of geogrid production and construction, systematic tests and analyses were conducted on their response characteristics before and after implantation and under different physical states. The demodulated optical signal of the fiber optic sensor before implantation is shown in Figure 10, while the optical signals of the sensing geogrid in the wound state after implantation and after being unrolled and laid on-site are shown in Figure 11.

Before implantation, the fiber optic sensor was demodulated. The demodulation results showed that the reflection spectrum of a single Bragg grating region presented a clear and isolated reflection peak, with the fiber optic sensor signal being continuous and uninterrupted.

After the sensing geogrid was packed and coiled, the optical signal exhibited significant attenuation at 40 m, but the signal was not lost. This indicates that the weaving process did not cause damage to the optical fiber sensors. The signal attenuation is mainly attributed to two mechanisms: (1) the small bending radius formed during the coiling of the sensing geogrid induced significant macro-bending loss [30] and (2) the self-weight of the sensing geogrid exerted compression on the internal sensors, leading to micro-bending deformation of the optical fibers, which disrupted the total internal reflection condition for light propagation within the fiber core, thereby generating additional micro-bending loss [31].

After the sensing geogrid was unfolded and laid out, the physical constraints were released, the optical signal intensity recovered, and the reflection peak characteristics became clear. This indicates that the optical fiber sensors were not permanently damaged, but rather experienced a temporary physical effect generated in the coiled state. The complete recovery of the sensing geogrid’s performance after unfolding demonstrates that it is capable of withstanding the mechanical actions during the production, transportation, and laying processes, possessing complete working performance in actual installation and meeting the requirements for subsequent monitoring.

### 3.2. Calibration of the Sensing Geogrid

**Figure 12:**
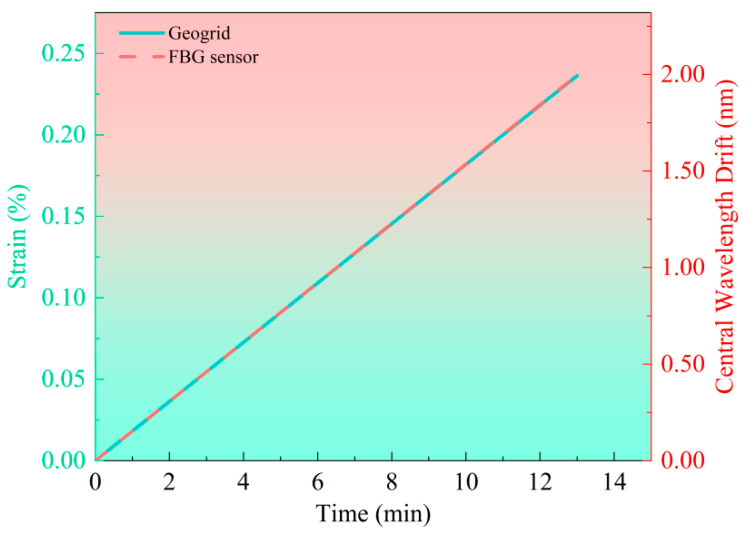
Relationship between sensor wavelength and geogrid strain.

The sensing geogrid was subjected to tension using a universal testing machine, and the sensor’s measured wavelength and the geogrid strain results were collected. The relationship curve is shown in Figure 12. This calibration result will be used to estimate the geogrid strain from field measurements.

As shown in Figure 10, a good linear relationship exists between the central wavelength shift of the fiber optic sensor and the axial strain of the geogrid. This indicates excellent deformation compatibility between the FBG sensor and the geogrid matrix, allowing it to reliably reflect the actual deformation state of the geogrid.

The longitudinal strain of the geogrid is defined by Equation (2):

(2) $$∆\varepsilon =\frac{∆l}{l_{0}}$$

where *Δε* is the longitudinal strain, Δ*l* is the increment of deformation within the initial gauge length, and *l*_0_ is the initial value of the initial gauge length.

Based on Equation (1) and the linear response between wavelength and strain, the following calibration relationship can be established:

(3) $$∆\varepsilon =\frac{1}{K_{\varepsilon }}∆\lambda_{BS}$$

Based on the experimental data, *K_ε_* is obtained through fitting, as shown in Equation (4). This coefficient will serve as a key parameter for converting wavelength signals into strain values during subsequent field monitoring.

(4) $$\frac{1}{K_{\varepsilon }}=845\;\mu \varepsilon /\mathrm{nm}$$

### 3.3. Wavelength Variation of the Sensor During Pavement Construction

In our previous study [29], temperature compensation tests were conducted under high-temperature conditions for the FBG sensors and their protective structure used in this study, and a corresponding temperature correction relationship was established. The results showed that, under the actual construction temperature conditions of this study, the equivalent strain error caused by temperature variations was approximately 0.02%, which was significantly lower than the magnitude of geogrid strain changes monitored during the construction process. Therefore, although temperature effects may have a certain influence on the optical fiber measurement results, their impact on the main conclusions of this study is relatively limited.

**Figure 13:**
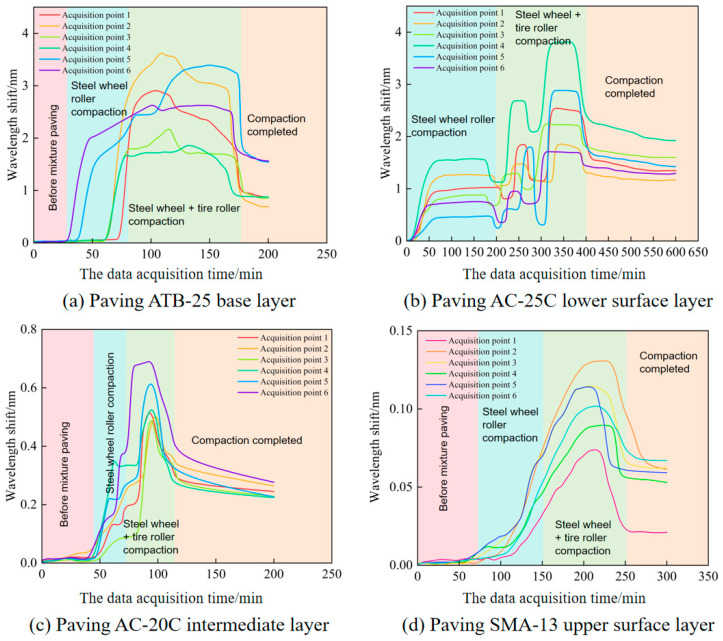
Time-history curves of wavelength variation with time at different sampling points.

In the asphalt pavement construction process, six representative sampling points were selected, and the time-history curves of wavelength variation with time at each sampling point are shown in Figure 13. Throughout the entire process in which the sensing geogrid was incorporated into the pavement structure (ATB-25 base course and AC-25, AC-20, and SMA-13 asphalt surface layers), the wavelength evolution of the optical fiber sensors exhibited a response pattern that was highly coordinated with the construction process, loading conditions, and temperature field. The monitoring data from each structural layer collectively reveal that the wavelength changes strictly followed the spatiotemporal sequence of the paving and rolling operations; the initial rolling consistently triggered a significant step-like jump in wavelength at all measurement points, confirming the dominant role of initial compaction in material densification and geogrid tensioning. As construction progressed, the wavelength response gradually shifted from being dominated by construction mechanics to multi-physics coupling: in the lower layer, the kneading effect of the tire roller was clearly manifested as a “rise-then-fall” fluctuation pattern, reflecting the transient process of local loosening and re-densification of the mixture; meanwhile, the plateau or slow decline stages appearing during the rolling intervals reflected the superposition effect of stress redistribution and early thermal relaxation. During the paving of the middle and upper layers, as the lower structure had hardened layer by layer and the transmission of construction machinery loads weakened, the wavelength changes shifted to be mainly controlled by the temperature field gradient of the asphalt mixture, presenting a differentiated increase related to the thermal diffusion rate. Throughout the construction period of the entire pavement structure, the dynamic history of the sensing geogrid’s wavelength changes—from bearing construction machinery loads to repetitive compaction and finally stabilizing—was completely recorded, validating the sensing system’s capability to distinguish between “construction loads–material behaviors–environmental actions.”

### 3.4. Strain Variation of the Geogrid During Pavement Construction

**Figure 14:**
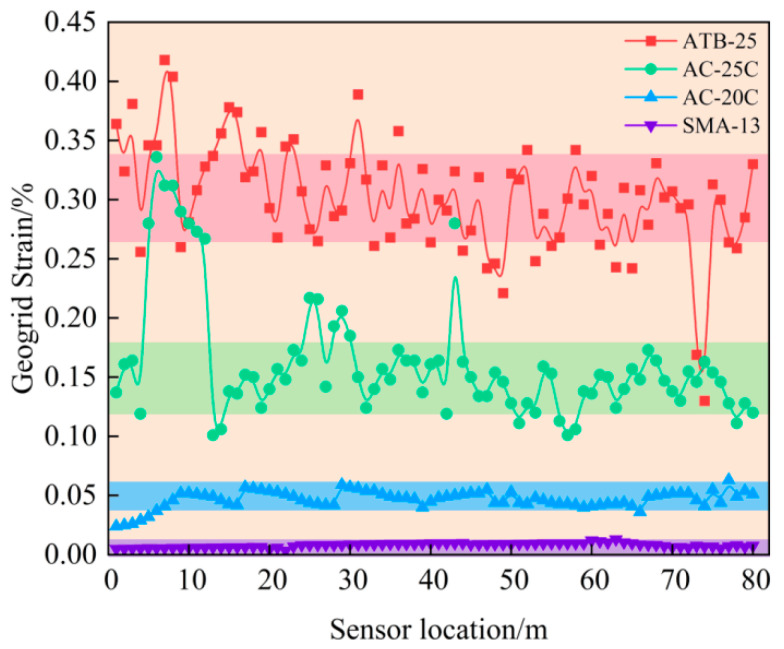
The maximum strain of the geogrid at each collection point during asphalt pavement paving.

During the paving process of the pavement structure (ATB-25 and asphalt surface layers AC-25C, AC-20C, and SMA-13), the maximum strain of the sensing geogrid at each measurement point exhibited a trend of decreasing layer by layer from the bottom up (Figure 14). In the ATB-25 base layer paving stage, the sensing geogrid was directly subjected to the rolling of construction machinery, and its strain response was the most significant, presenting large fluctuations. The maximum strain ranged between 0.418% and 0.130%, with the maximum strain at each measurement point occurring during the operation of the tire roller, as the sensing geogrid was significantly affected by construction disturbances during this stage. During the AC-25 lower layer paving stage, due to the isolation and stress diffusion effects of the base layer, the disturbance of construction machinery on the sensing geogrid weakened, and the strain fluctuation range decreased. When paving the AC-20 middle layer, the strain further decreased, and the changes tended to stabilize; however, influenced by the local flatness differences of the geogrid left over from the base layer paving, the strain response manifested as segmented stepwise fluctuation characteristics. When paving reached the top layer SMA-13, the strain of the sensing geogrid approached the zero baseline, indicating that the disturbance influence depth of construction machinery was mainly concentrated within the range of about 22 cm from the base layer to the middle layer, while the mechanical influence of the top layer paving on the deep-seated sensing geogrid was already very limited. Overall, the attenuation process of the sensing geogrid’s maximum strain from the base layer to the top layer revealed the “mechanical shielding” and stress diffusion effects formed by the layer-by-layer hardening of the pavement structure, as well as the transmission pattern where construction disturbances significantly weaken with increasing depth.

### 3.5. Relationship Between Compaction Machinery and Optical Fiber Sensor Wavelength/Geogrid Strain

**Figure 15:**
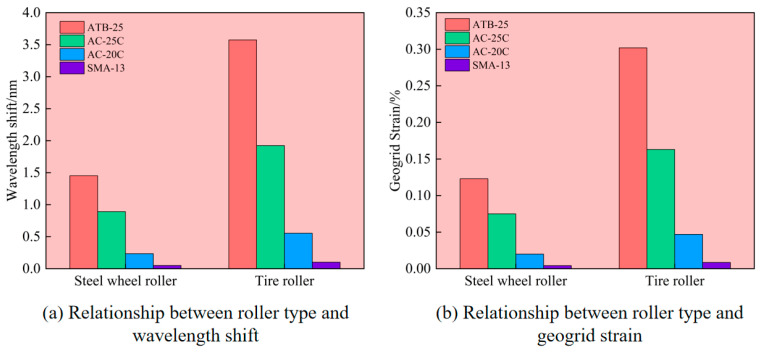
Relationship between compaction machinery and optical fiber sensor wavelength/geogrid strain.

During different construction stages of the asphalt surface layers, the effects of the steel wheel roller and the tire roller on wavelength shift present significant differences. According to the measured data from various sampling points, the average values of wavelength shift and geogrid strain caused by a single operation of the two types of compaction machinery are shown in Figure 15. During the paving process of the asphalt pavement structure, the sensing geogrid strain and the corresponding optical fiber wavelength shift induced by a single rolling pass of the roller exhibit a decreasing trend with the increase in asphalt coverage thickness, and significant differences exist in the influence of different compaction equipment. Since the axle load of the tire roller (30 t) is far greater than that of the steel wheel roller (10 t), the mechanical action it exerts on the geogrid is stronger, making it the primary source of wavelength shift and geogrid strain. In the construction of each structural layer, the variation caused by the steel wheel roller is only between 41% and 50% of that caused by the tire roller. This is because the self-weights of the two differ significantly, and their compaction mechanisms are not entirely the same: the steel wheel roller performs preliminary compaction through vibration and impact, while the tire roller has a larger contact area and horizontal kneading effect that makes the asphalt mixture denser, thereby causing more significant tensile deformation of the sensing geogrid during the construction process. With the increase in paving thickness, the lower structure of the pavement hardens, and the overall stiffness improves, making the attenuation of construction load transmission more obvious; therefore, the strain responses induced by both types of equipment weaken layer by layer.

### 3.6. FWD Test

**Figure 16:**
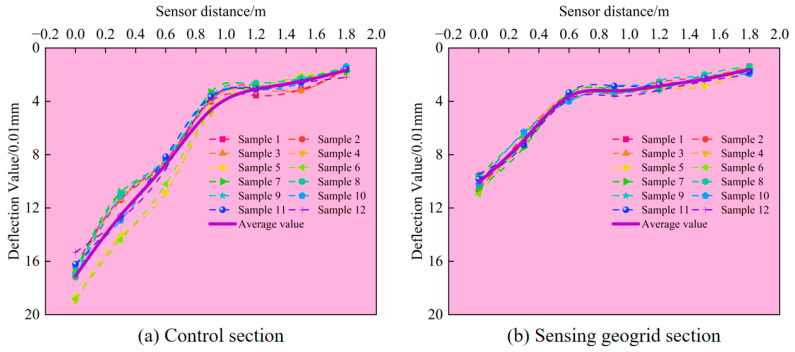
Deflection basin curves for different pavement structures.

After the completion of pavement paving, FWD tests were conducted on the sensing geogrid section and the control section, with the deflection basin test results shown in Figure 16. The results indicate that the shapes of the deflection basin curves for the two pavement structures are similar, with deflection values gradually decreasing as the distance from the load center increases. The deflection curve of the ordinary pavement structure has a larger slope, and the deflection attenuation is significant within the range of 0 to 0.9 m from the load center, with a maximum deflection value of 0.17 mm. The deflection curve of the pavement structure reinforced with the sensing geogrid is relatively gentle; the deflection attenuation is relatively obvious within the range of 0 to 0.6 m from the load center, and the maximum deflection value is 0.10 mm, representing a 41% reduction in pavement deflection. During the load transmission process, the sensing geogrid acts to diffuse stress, delaying the development of deflection. Meanwhile, the sensor-equipped geogrid has relatively high strength. The transverse ribs of the geogrid limit the lateral movement of particles in the surface or base layers, while the longitudinal ribs, together with the transverse ribs, form an integral mesh, providing lateral restraint to the pavement structure and enhancing interlayer stiffness. When vehicle loads are applied to the pavement, the geogrid can distribute stresses and improve interlayer cooperation, thereby increasing the overall stiffness and deformation resistance of the pavement and delaying the propagation of reflective cracks.

Table 2 presents the statistical analysis of the FWD test results for the conventional section and the sensor-equipped geogrid-reinforced section. Overall, the deflection values of both sections gradually decreased with increasing distance from the load center, indicating a consistent load distribution pattern within the pavement structure. However, within the range of 0–0.9 m, the mean deflection values of the sensor-equipped geogrid-reinforced section were 23.1–59.7% lower than those of the conventional section, and all differences were highly significant (*p* < 0.001). This indicates that geogrid reinforcement significantly improved the overall stiffness of the pavement structure and enhanced its load transfer capability. The coefficients of variation (CVs) for both sections were less than 13%, demonstrating good stability and repeatability of the test data. Moreover, the overall CV values of the sensor-equipped geogrid-reinforced section were lower than those of the conventional section, indicating that geogrid reinforcement can effectively homogenize the stress distribution within the structure and reduce the variability of local deformation. As the distance from the load center increased (1.2–1.8 m), the differences in deflection between the two sections gradually diminished (*p* > 0.05), suggesting that the reinforcement effect of the geogrid was primarily concentrated within the core loading zone of the FWD.

## 4. Conclusions

To address the difficulty of obtaining real-time information on internal stresses and deformations during the reinforcement of asphalt pavements with conventional geogrids, this study applied FBG-based sensor-equipped geogrids to the Qugang Expressway project in Hebei Province. The sensing performance, constructability, and pavement reinforcement effectiveness of the geogrids were systematically evaluated through both laboratory tests and field monitoring. The main conclusions are summarized as follows:

(1) Optical fiber signal testing indicates that the weaving process did not cause structural damage to the FBG sensors. Although the sensing geogrid experienced temporary optical signal attenuation due to micro-bending and macro-bending effects during coiled transportation, the signal returned to normal after being unfolded and laid on-site, demonstrating that the sensing geogrid possesses stable working performance.
(2) The central wavelength shift of the optical fiber sensor presents a good linear relationship with the axial strain of the geogrid, the strain transfer coefficient is stable, and there is excellent deformation compatibility between the two.
(3) During the layer-by-layer paving process of the asphalt pavement structure, the sensing geogrid was able to completely capture the mechanical responses at each stage. The strain response decayed layer by layer from bottom to top: the disturbance was greatest during base layer paving, with significant strain fluctuations; as the middle base layer hardened to form “mechanical shielding,” the strain response in the middle and lower layers gradually weakened; the influence was negligible during upper layer paving, with the significant influence depth of construction machinery being approximately 22 cm.
(4) Due to its heavy axle load and static kneading effect, the strain induced by the tire roller during a single pass was approximately twice that of the steel wheel roller, serving as the main source of geogrid deformation during the construction period. With the increase in pavement structure thickness, the overall road stiffness improved, causing attenuation in the transmission of construction loads, and the strain responses induced by both types of rollers decreased layer by layer.
(5) The maximum deflection of the section reinforced with the sensing geogrid was approximately 41% lower than that of the control section, and the FWD deflection basin was relatively flatter, with a significant reduction in deflection within the range of 0–0.9 m. The geogrid enhanced the overall stiffness and load distribution capacity of the pavement and reduced the variability of local deformation within the test range. The results indicate that the geogrid can improve the mechanical response of the pavement; however, its actual effect on the long-term development of reflective cracking still requires further monitoring and verification.

In summary, this study successfully fabricated a sensing geogrid with both reinforcement and sensing functions through the weaving process. It is capable of coordinating deformation with the pavement structure and accurately monitoring the mechanical response during the construction process. Its long-term performance, particularly the effect of delaying reflective cracks, remains to be verified through continuous monitoring.

## Figures and Tables

**Table 1:** Physical and mechanical properties of the composite geogrid.

Index		
ultimate tensile strength (kN/m)	transverse	80
	longitudinal	50
ultimate elongation (%)	transverse	≤2
	longitudinal	≤3
Thickness (mm)		0.6
aperture size (mm × mm)		25 × 25

**Table 2:** Statistical analysis of FWD test results.

Sensor Location	Control Section			Sensing Geogrid Section			*p*-Value
	Mean	SD	CV	Mean	SD	CV	
0	17.11	1.15	6.71	10.09	0.51	5.07	< 0.001
0.3	12.32	1.36	11.01	6.90	0.40	5.76	< 0.001
0.6	9.01	1.02	11.32	3.63	0.22	6.18	< 0.001
0.9	3.98	0.49	12.33	3.21	0.24	7.34	< 0.001
1.2	3.00	0.23	7.73	2.86	0.22	7.70	0.123
1.5	2.58	0.39	15.14	2.34	0.27	11.50	0.094
1.8	1.66	0.23	14.02	1.60	0.20	12.44	0.505

## Data Availability

The original contributions presented in this study are included in the article. Further inquiries can be directed to the corresponding authors.

## Abbreviations

The following abbreviations are used in this manuscript:

FBG	Fiber Bragg grating
ATB-25	Asphalt-treated base
CSM	Cement-stabilized macadam
FWD	Falling weight deflectometer
